# Replacement of milk fat by rapeseed oil stabilised emulsion in commercial yogurt

**DOI:** 10.7717/peerj.16441

**Published:** 2023-12-11

**Authors:** Mirosław M. Kasprzak, Marek Sady, Joanna Kruk, Simona Bartkova, Immanuel Sanka, Ott Scheler, Ewelina Jamróz, Wiktor Berski, Sylwia Onacik-Gür, Rafał Szram, Charles Odilichukwu R. Okpala, Joanna Tkaczewska, Marzena Zając, Jacek Domagała, Stanisław Ptasznik

**Affiliations:** 1 Department of Animal Product Processing, Faculty of Food Technology, University of Agriculture, Cracow, Poland; 2 Department of Engineering and Machinery for Food Industry, Faculty of Food Technology, University of Agriculture, Cracow, Poland; 3 Department of Chemistry and Biotechnology, Tallinn University of Technology, Tallinn, Estonia; 4 Department of Chemistry, University of Agriculture, Cracow, Poland; 5 Department of Carbohydrates Technology and Cereals Processing, Faculty of Food Technology, University of Agriculture, Cracow, Poland; 6 Department of Meat and Fat Technology, Prof. Wacław Dąbrowski Institute of Agricultural and Food Biotechnology-State Research Institute, Warsaw, Poland; 7 UGA Cooperative Extension, University of Georgia, Athens, Georgia, United States; 8 Faculty of Biotechnology and Food Sciences, Wroclaw University of Environmental and Life Sciences, Wroclaw, Poland

**Keywords:** Quality lipid replacement, Emulsion structure, Image analysis, Sensory analysis, Yogurt

## Abstract

The incorporation of lipid droplets and further characterization of matrices within dairy products may be possible using such adjacent particles as protein complexes/lipids. Among the range of varied emulsions and their functionalities, great attention has recently focused on the fabrication of high internal phase types. Feasibly, stable alternatives structured with health-beneficial lipids like those derived from plants could replace saturated fatty acids. As a fat replacement strategy, the fate of incorporated HIPE would require some adjustments either with storage stability and/or structural feat for the food matrix. Therefore, the replacement of milk fat by rapeseed oil stabilised emulsion in commercial yogurt was investigated. This involved 25%, 50% and 75% rapeseed oil respectively assigned as low (LIPE), medium (MIPE), and high internal phase emulsion (HIPE). Specifically, emulsions were examined by droplet size, encapsulation, pH, zeta potential, phase separation, and rheology. The fat free yogurt supplemented by HIPE were examined by droplet size, zeta potential, pH, color, sensory, texture and microbiological aspects against positive (regular milk fat) and negative (fat free) yogurt controls. Results showed increasing rapeseed oil contents would form smaller droplet-like emulsions. Within the yogurt matrix however, incorporating HIPE would seemingly reduce oil droplet size without much compromise to bacterial viability, sensory, or texture. Overall, this simple method of lipid alternation shows promise in dairy products.

## Introduction

The global food industry appears to be moving more towards the development and production of plant-based alternatives to traditional animal-based foods (Alcorta et al., 2021). In particular, this rising demand for plant-based products has facilitated the need for improved approaches to create foods that not only have attractive appearance, flavour, and texture but also provide balanced nutrition values (McClements & Grossmann, 2021). As the body of evidence that links high consumption of saturated lipids with cardiovascular disease events in adults continues to accumulate (Mozaffarian, Micha & Wallace, 2010), to incorporate the likes of unsaturated lipids especially those obtained from plants so as to alleviate the negative health conditions such as overweight or obesity (Clark, Pope & Belarmino, 2022) has become increasingly sought after. For instance, the replacement of dairy fat with rapeseed oil would produce rapid improvements in hyperlipidemia has been reported (Iggman et al., 2011). However, to efficiently encapsulate the targeted oils and at the same time, maintain their stability remains a challenge especially within liquid or semi-liquid food products when replacing saturated fatty acids or partial substitution with unsaturated fatty acids. Structuring the liquid oil through emulsification could be a promising physical method for replacing milk fat (Kupikowska-Stobba & Kasprzak, 2021), which would be successfully attainable through the formulation of emulsions through the use of either low- or high-energy methods. In particular, the high-energy methods would employ the use of high shear forces to disrupt the internal phase into droplets, whereas the low-energy methods would rely on the changes in chemical potential of emulsion constituents that occurs as a result of changes in composition or temperature, which allows for the production of emulsions through gentle mixing (Maali & Mosavian, 2012). The production of oil-in-water emulsions involves the use of two immiscible phases, where one phase is encapsulated in the form of droplets within the other phase. Being thermodynamically unstable (with an exception of micro-emulsions), the added emulsifiers are adsorbed at the interface that leads to the entrapment of the internal phase in a form of droplets in the continuous emulsion phase. Among the range of varied emulsions and functionalities, a great attention has been recently focused on the fabrication of high internal phase emulsions (HIPE) with a content of internal phase being greater than 74% (Vélez-Erazo et al., 2021). Stable alternatives structured with health beneficial lipids, like those derived from plants, could further replace saturated fatty acids. Like a fat replacement strategy, the fate of incorporated HIPE would require some adjustments, either with storage stability and/or structural feat for food matrix. Typically, the characterization of lipid droplets in emulsions would be achieved *via* such approaches as light scattering, microscopic image analysis, ultrasonic sizing, and nuclear magnetic resonance (Linke, Weiss & Kohlus, 2020; Silva et al., 2022; Kostoglou et al., 2022). Of these methods, light scattering techniques appear frequently despite limitations in detecting as well as differentiating oil droplets from irregular protein particles within food matrices. To overcome this limitation, the use of image analysis utilizing advanced logarithmic capabilities by accurately distinguishing the round structure of oil droplets from irregular protein complexes may help. Indeed, there is paucity of relevant information regards the incorporation of lipid droplets and further characterization of matrices within dairy products that possess varied morphology, like protein complexes/lipids. To supplement existing literature, therefore, the replacement of milk fat by rapeseed oil stabilised emulsion in commercial yogurt was investigated. This involved 25%, 50% and 75% rapeseed oil respectively assigned as low (LIPE), medium (MIPE), and high internal phase emulsion (HIPE). Specifically, emulsions were examined by droplet size, encapsulation, pH, zeta potential, phase separation, and rheology. The fat free yogurt supplemented by HIPE were examined by droplet size, zeta potential, pH, color, sensory, texture and microbiological aspects against positive (regular milk fat) and negative (fat free) yogurt controls.

## Materials and Methods

### Schematic overview of the experimental program

**Figure 1:**
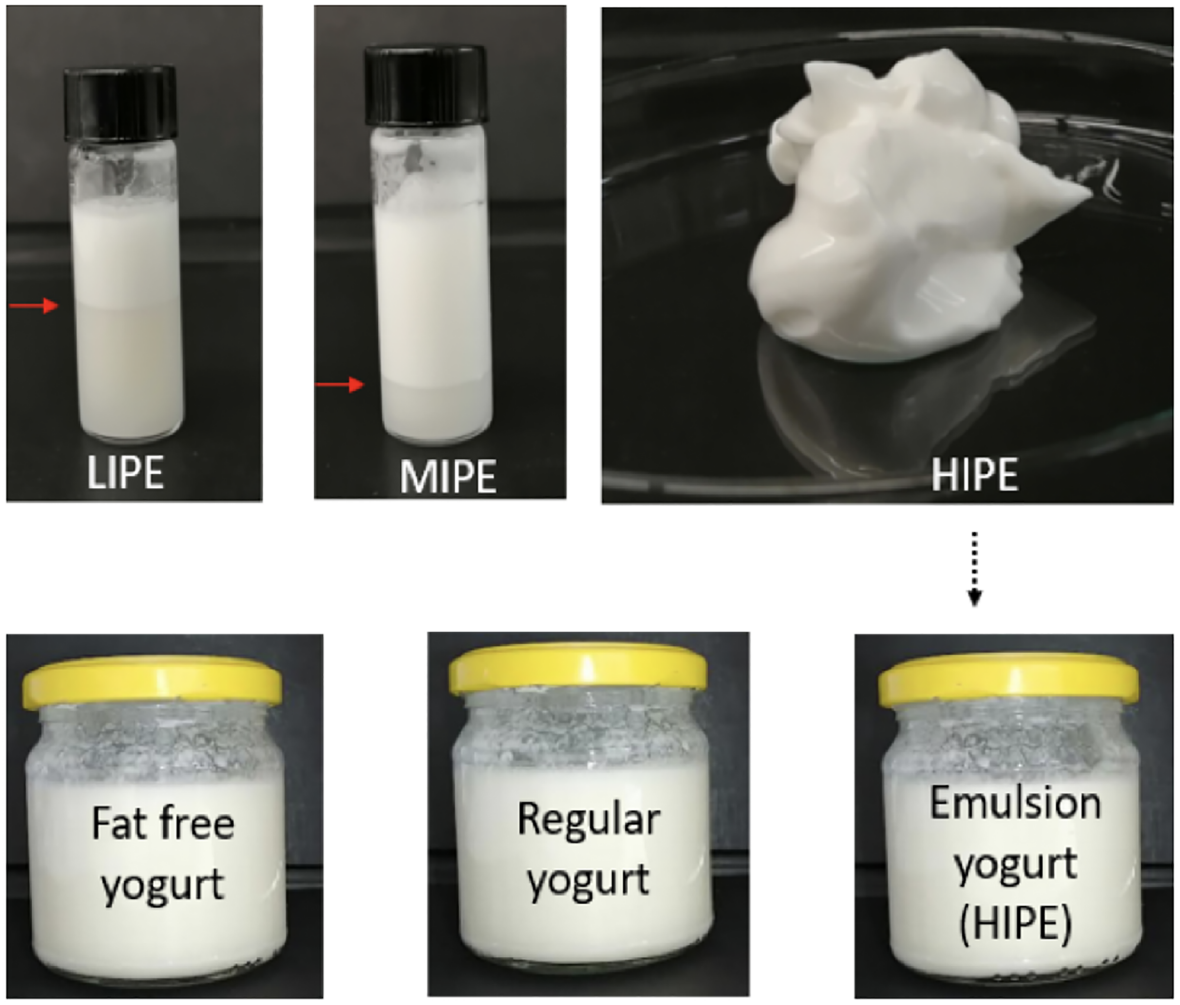
Schematic overview of experiment. LIPE, low internal phase emulsion; MIPE, medium internal phase emulsion; HIPE, high internal phase emulsion. Pointer (red arrow) indicated the phase separation in the LIPE and MIPE.

The schematic overview of the experimental program is shown in Fig. 1, which depicted the tested LIPE, MIPE, and HIPE and fat free yogurt (0.0% lipids), regular yogurt (2.5% of milk fat), and emulsion yogurt (2.5% rapeseed oil). As a consequence of no phase separation of HIPE, this emulsion was then incorporated into the matrix of commercial stirred fat free natural yogurt for investigation of texture, sensory, and microbiological properties along with structural oil droplet measurement by the image processing software that distinguishes the round structure of the oil droplets from irregular protein complexes in yogurt matrix. The experimental procedures were conducted in adherence to laboratory guidelines set out by the Department of Animal Product Processing, Faculty of Food Technology, University of Agriculture, Cracow, Poland.

### Assembly of experimental materials

Rapeseed oil (trade name “Kujawski”, Bunge Polska Sp. z o.o., Kruszwica, Poland) was purchased from a local retailer. Whey protein concentrate traded as Whey Protein Concentrate 80 (protein content 74.1%) was obtained from SM Mlekovita (Wysokie Mazowieckie, Poland). Natural stirred yogurts, traded as a fat free and a regular natural drinking yogurt were obtained from a dairy cooperative (Cuiavia, Inowrocław, Poland). As the manufacturer reported that regular, full fat yogurt contained of 2.5% lipids (including 1.6% saturated lipids), 4.0% protein, 6.0% of carbohydrates (including 5.0% sugars) and 0.04% salt. Free fat yogurt had 0.0% lipids, 4.0% protein, 6.0% of carbohydrates (including 5.0% sugars), and 0.04% salt. The pH values of produced emulsions were monitored by a pH meter (CP-505 electrode; El-metron, Zabrze, Poland).

### Manufacture of emulsions

A day before formulation of emulsions, dispersion of 5,9% whey protein was formulated in milli-Q water by a shear mixing at 20,000 rpm for 4 min and remained at 4 ±1 °C overnight in order to fully hydrate. The rapeseed oil was added to a formulated dispersion and mixed by homogenisation at 25,000 rpm for 5 min. The high shear rate homogenizer (Unidrive × 1000; Ingenieurbüro Cat, Ballrechten-Dottingen, Germany) was used. The added content of oil was at 25%, 50% or 75% (w/w basis) in a total weight of LIPE, MIPE and HIPE, respectively.

### Incorporation of emulsion as a lipid carrier

The HIPE was further selected as a carrier of rapeseed oil and was incorporated into the yogurt matrix. An appropriate amount of HIPE was added to fat free yogurt, followed by mixing at 15,000 rpm for 60 s by homogenizer (Unidrive × 1000; Ingenieurbüro Cat, Ballrechten-Dottingen, Germany) in order to enrich yogurt with 2.5% rapeseed oil (equal to milk fat in regular yogurt). This processed yogurt was later referred to as the emulsion yogurt. Similarly, the free fat yogurt alone and regular yogurt were mixed as above to introduce the same physical changes to the matrix base. Prior to color, viscosity, and texture analysis, all three yogurts were stored in the fridge at 4 ± 1 °C overnight in order to allow the re-build of the food matrix.

### Analytical measurements

#### Composition of whey protein concentrate

The composition of whey protein concentrates specific to protein content and dry matter (DM) was determined by AOAC method (AOAC International, Horwitz & Latimer, 2007). The fat content was determined by the Soxhlet method as described by Petrović, Savić & Petronijević (2016).

#### Emulsions encapsulation efficiency and emulsification index

The emulsion encapsulation efficiency by loss of oil (LO) was measured on days 1, 7, and 30. At each of the individual time points, a sample of 1.0 g was weighted into Eppendorf tube (2.0 mL) and centrifuged at 10,000 rpm for 30 min at 4 °C as described by Vélez-Erazo et al. (2021). After the centrifugation, the amount of the expelled oil has been removed, which has allowed the weighing of remaining mass, followed by the calculation of oil loss, as shown by the Eq. (1) below:

(1) $$LO = {{\left( {{m_i} - {m_f}} \right)} \over {\left( {{m_i} - m} \right)}} \times 100 \%$$

where, m_i_ is the mass of sample including an Eppendorf tube, m_f_ is the mass of sample and Eppendorf after a removal of free oil, whereas m is the Eppendorf mass alone. The emulsification index (EI) employed a phase separation approach as previously described by Kannan et al. (2017) with slight modifications. In order to establish the EI, a quantified volume of 7 mL emulsion was filled into 10 mL glass vials, and subsequently kept for a 30 day period. The emulsification index (EI) was calculated as total height of the emulsified layer (H_e_) divided by total height of the liquid column (H_t_) as shown by the Eq. (2) below:

(2) $$EI = {{He} \over {Ht}} \times 100 \%$$

#### Microstructure and image assessment

**Figure 2:**
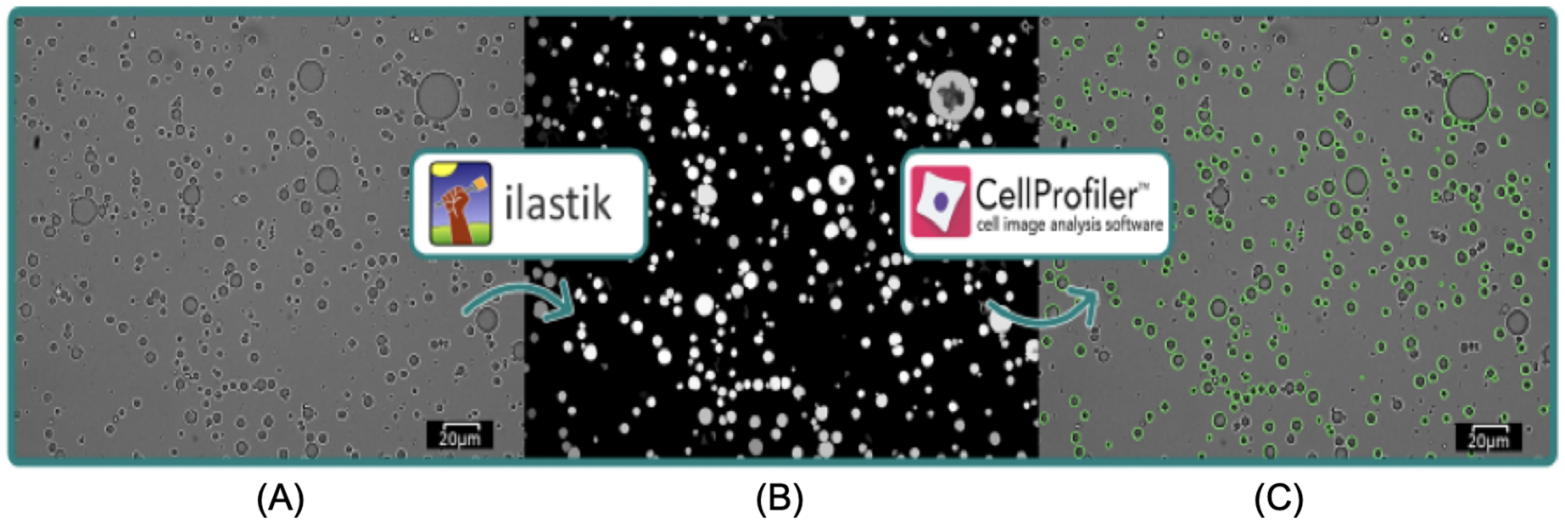
Visualization of image analysis workflow with ilastik and CellProfilerTM. Scale bar: 20 µm. Raw image (A) was imported into ilastik, which produced a probability map (B) that was further imported into CellProfilerTM, where emulsion droplets were re-detected as illustrated by green outlines (C).

The microscopic structures of emulsions and yogurts were visualised using a light microscope (Olympus BX61 microscope with PlanApo N 60x oil objective lens). In order to identify the oil droplets in yogurts (rich in protein complexes), a drop of emulsion was placed onto a glass slide and stained with a marker for lipids (Sudan III; WarChem, Warsaw, Poland), followed by sliding over a glass cover slip. A minimum of 24 images were taken per sample and saved in tagged image file (TIF) format. All images were subjected to processing analysis by open-source software CellProfiler^TM^ (version 4.2.1) (Stirling et al., 2021) and ilastik (version 1.3.3) (Berg et al., 2019). Ilastik was used to detect and provide a probability map representing the emulsions in each image. This was done using a previously published workflow by Sanka et al. (2021b), with sigma or scale value set to 0.30, 1.00, and 3.50. In short, five randomly selected images from each sample were imported into Ilastik and emulsions were labelled as objects of interest, while everything else was labelled as background. This was used to train Illastik for determining the objects of interest (*i.e*., the emulsion droplets) in the rest of the sample images. Thresholding method was set to hysteresis, with core value 0.85 and final values between 0.45–0.40 depending on the sample. A second classification enabled removal of any possible incorrectly detected objects. Finally, a probability map was created in the Object Information Export module that was subsequently imported into CellProfiler^TM^. The emulsion droplets were re-detected in “Identify Primary Object” module using the probability maps (Fig. 2), as this results in higher emulsion detection rate and less errors than direct detection from brightfield images. Further settings for processing are described in previous work (Sanka et al., 2021a). Finally, individual emulsion droplet size measurements were exported as.csv files. Each of the yogurt samples were calculated from minimum 140 droplets and the emulsion samples from minimum 4,320 droplets. The size distribution of oil droplets and droplet mean diameter D_[3,2]_ of the emulsion D_[3,2]_ were calculated according to the following Eq. (3):

(3) $${D_{\left[ {3,2} \right]}} = \left( {\sum {n_i}d_i^3} \right)/\left( {\sum {n_i}d_i^2} \right)$$

where, d_i_ is the diameter of droplet, n the number of droplets.

Furthermore, the resulted droplets sizes were reported as D_[10]_, D_[50]_, D_[90]_. The parameter related to the width of size distribution was expressed according to Eq. (4):

(4) $$Span = \left( {{d_{\left[ {90} \right]}} - {d_{\left[ {10} \right]}}} \right)/{d_{\left[ {50} \right]}}$$

where d_[10]_, d_[50]_, d_[90]_ are the equivalent volume diameters at 10%, 50% and 90% of cumulative volume, respectively.

#### ζ-potential measurements

The ζ-potential of emulsions and yogurts were measured using Zetasizer (Nano ZN Malvern, USA). The samples were diluted by 1,000 folds with milli-Q water before the measurements.

#### Rheology

Rheological characterization of tested systems was performed with the use of a rotational rheometer RS 6000 (Haake, Karlsruhe, Germany). A cone-plate measuring system was used (diameter 60 mm, angle 1°) with a measuring gap 0.5 mm. Apparent viscosity of emulsions and yogurts was measured by raise of shear rate from 1 to 300 s^−1^, over a 300 s period and a subsequent decrease of shear rate from 300 to 1 s^−1^ (hysteresis loop test), over a 300 s at temperature 20 (±0.1) °C. Obtained flow curves were described by Ostwald de Waele rheological model according to Eq. (5):

(5) $$\tau = K\cdot{\dot \gamma ^n}$$

where: τ–shear stress (Pa), K–consistency coefficient (Pa·s^n^), $\dot \gamma$–shear rate (s^−1^), and *n*–flow behavior index.

The data obtained from the hysteresis loop test were used to determine the energy dissipated by the sample according Eq. (6):

(6) $$E = P\cdot \Delta t\cdot V$$

where: $P$–surface area between the flow curves up and down, Pa·s^−1^; $t$–measurement time, s; $V$–volume of sample, m^3^.

Viscoelastic properties were examined using oscillatory tests. Storage (G’) and loss (G”) modulus were measured in the deformation ( $\gamma$) range from 0.001 to 100 at selected frequency 1 Hz. The measuring geometry, gap and temperature was the same as the determination of flow curves. Measurements were carried out in triplicates.

#### Texture measurement

With all yogurts in cylindrical glass jars (with a dimension of 60 mm internal diameter and 70 mm high) weighted 110 g with average (product) height of 40 ± 5 mm, the texture measurements were determined under the sample temperature of 4 (±1) °C using a back-extrusion. Texture considerations specific to penetration tests employed Texture Analyser TA-XT plus (Stable Micro Systems, Surrey, UK), which was probed with diameter of 40 and 5 mm of width to deliver a penetration depth of 20 mm and rate of 1 mm/s. The computer software (Texture Exponent v. 2.0.7.0.) connected to the Texture Analyser enabled the output diagrams of force dependence on time, and produced data outputs of cohesiveness [N], consistency [N.mm] and firmness [N].

#### Color measurements

Color of emulsions and yogurts were determined in the CIE Lab system by an instrumental colorimeter (CM-3500d; Konica Minolta, Osaka, Japan). Prior to the measurements, the instrument was calibrated with white and black enamel according to the producer’s instructions. A fixed amount of sample was poured into the measurement cell. The readings were collected by the reflectance mode, illuminant D65 and observer angle 10°. Color was represented by L* values for lightness, a* for +a is the red direction, -a is the green direction, and b* values, for +b is the yellow direction, and -b is the blue direction. In addition, the total color difference (ΔE) was calculated to highlight comparative differences between samples by Eq. (6):

(7) $$\Delta E = {\left( {\Delta{L^2} + \Delta{a^2} + \Delta{b^2}} \right)^{1/2}}$$

where, ΔL, Δa and Δb are the differences in the specified tristimulus coordinate between the sample and a reference.

#### Microbial determinations

The analysis was carried out in accordance with the general requirements for microbial evaluation by ISO 7218 (2007), using the decimal dilutions of the sample that were prepared according to ISO 6887-1 (2017). Buffered Peptone Water (Oxoid, Basingstoke, UK) was used as the diluent. Enumeration of *Lactobacillus delbrueckii subsp. bulgaricus* and *Streptococcus thermophilus* were performed according to PN (ISO 7889, 2007) using MRS LAB-AGAR^TM^ media (BioMaxima, Lublin, Poland), the pH of which was adjusted to 5.4 by the addition of anhydrous acetic acid (Chempur, Piekary Śląskie, Poland) and M17 AGAR (Oxoid Limited,, Basingstoke, UK) pH 6.8 adjusted with 0.1 M HCl (Stanlab, Lublin, Poland). The anaerobic conditions required for the cultivation of *L. delbruecki ssp. bulgaricus* were provided using anaerobic jar (schuett-biotec GmbH, Gottingen, Germany). The number of yeasts and moulds was determined according to ISO 21527-1 (2008), using DRBC LAB-AGAR^TM^ medium (BioMaxima, Lublin, Poland).

#### Sensory evaluation

Sensory evaluation was conducted with the help of 14 trained panellists who tested three samples of yogurts such as emulsion yogurt, fat free and regular yogurt. The panellists received cards with detailed descriptions of the typical characteristics of yogurts and samples of 100 g each with a three-digit random code, with a modified method by Baryłko-Pikielna & Matuszewska (2014). The following quality descriptors and corresponding importance factors were taken into accounts in 5-point scale analysis (where number 1 indicated “worst” and number 5 Indicated “best” category for the given product): color—0.10, whey exudate—0.10, texture—0.25, smell—0.20 and taste—0.35. The descriptor of overall sensory quality for a product was evaluated using the importance factor for individual quality descriptors. In addition, the samples were evaluated using a nine-point hedonic scale (1—“highly disagree” and 9—“liked very much”) for the attributes of appearance and color, smell, taste, texture and overall acceptance. In order to minimize the inaccuracy and masking of sensory qualities, water was offered to rise after the taste of samples.

### Statistical analysis

Data arising from triplicate determinations were submitted to one-way analysis of variance (ANOVA). The probability level to detect statistical significance was set at *p* < 0.05. Mean comparison employed Tukey *post-hoc* test. Statistica 12.5 (Tibco, Palo Alto, CA, USA) was used to run the data.

## Results and Discussion

The chemical composition analysis of whey protein concentrate revealed that it consisted of 74.1% protein and 3.2% lipids. In order to evaluate its emulsifying capabilities, whey protein concentrate at 5.9% of pure protein was utilised to stabilise different percentages of rapeseed oil (25%, 50%, and 75%, w/w) in a standardized emulsification process. The stability of the resulting emulsions was evaluated by examining their integrity in storage vials at days 1 and 30 after processing. The results indicated that whey protein was able to effectively emulsify all tested levels of added oil, with no signs of coalescence observed after 30 days of storage, regardless of the oil content.

### Mean diameter, size distribution and rheology of emulsions

**Figure 3:**
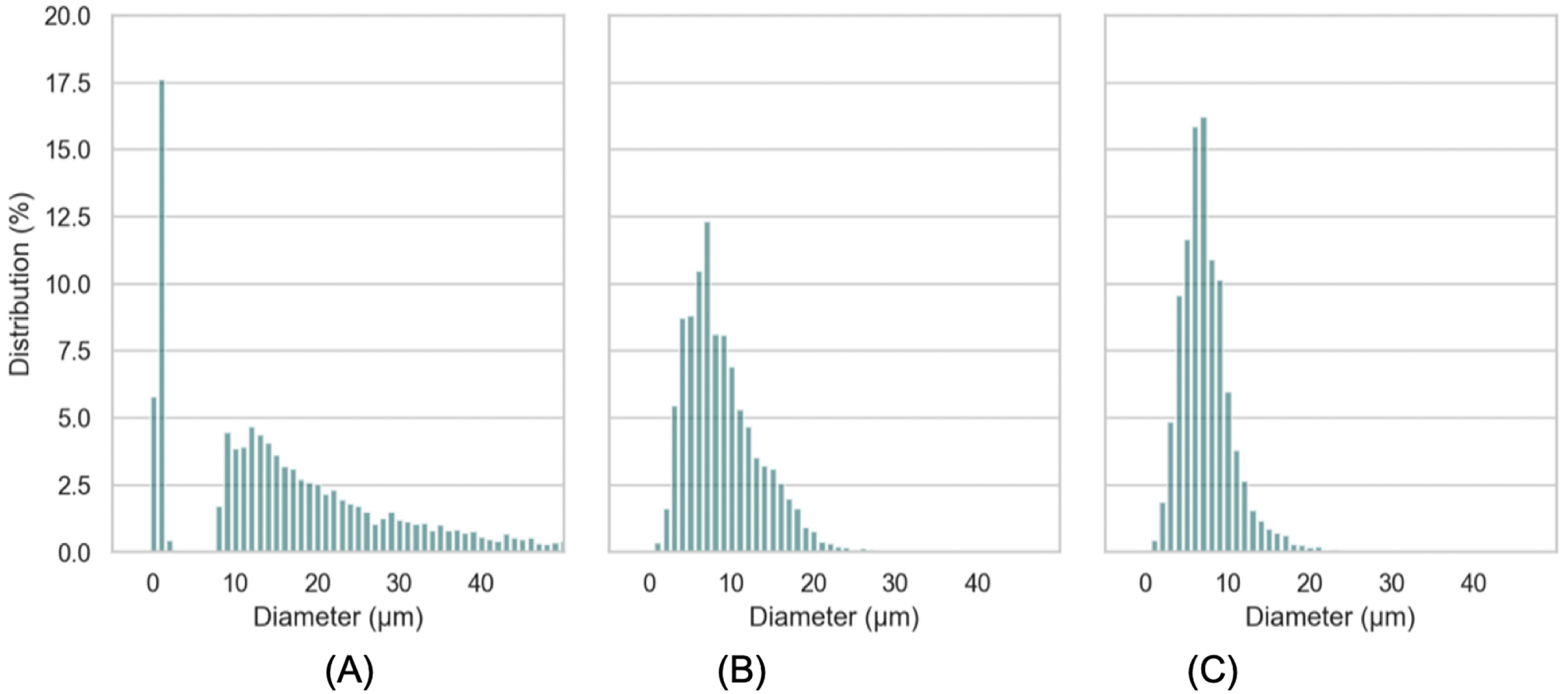
Distribution of droplet size of LIPE (A), MIPE (B), HIPE (C). LIPE, low internal phase emulsion; MIPE, medium internal phase emulsion; HIPE, high internal phase emulsion.

**Figure 4:**
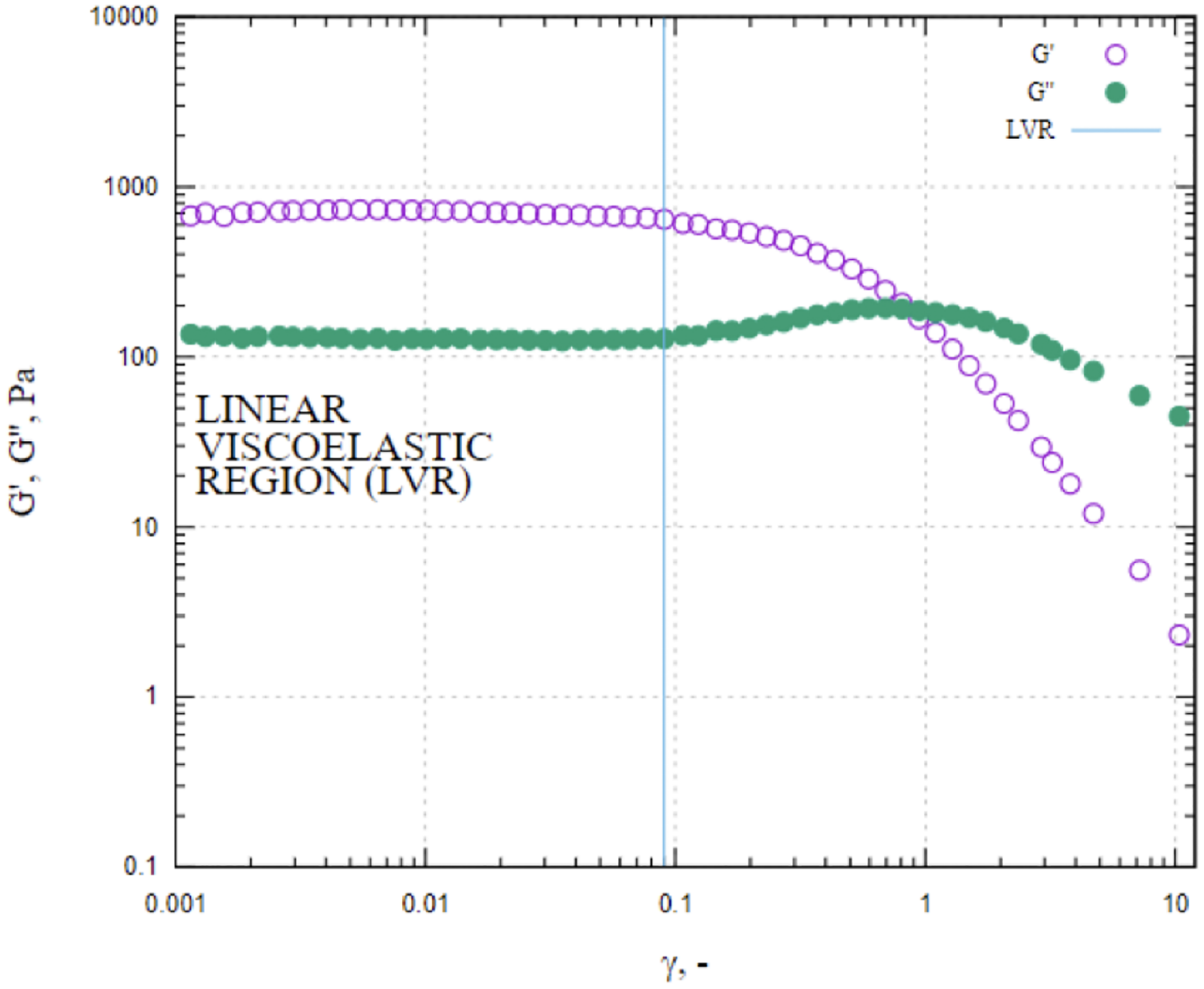
Values of storage and loss moduli (G′ and G″) of HIPE measured in stress sweep test. HIPE, high internal phase emulsion

The macroscopic characteristics of an emulsion can have a significant impact on the stability, organoleptic properties and texture of the final product (Jie & Chen, 2022). A variety of factors, including the concentration and type of emulsifiers, the ratio of internal and external phases, the conditions of homogenization and the viscosity of the constituents, can influence the droplet size of the emulsion (McClements, 2004b). In this work, characteristics of droplet size of LIPE, MIPE and HIPE are shown in Table 1. The emulsions produced with 25%, 50%, and 75% rapeseed oil demonstrated mean droplet size 17.0 ± 13.39, 9.3 ± 4.54 and 5.5 ± 2.53 µm (*i.e*., LIPE, MIPE and HIPE), respectively. The LIPE showed bimodal distribution, whereas HIPE had a monomodal distribution (Fig. 3). More so, 50% oil rich emulsion showed single mode distribution with a long shoulder towards a greater size of oil droplets. Table 2 shows the characteristics of droplet size in yogurt from the context of lipid contents. Cortés-Muñoz, Chevalier-Lucia & Dumay (2009) reported increasing content of oil at 15%, 30% and 45% on emulsions droplet size by mixing at 5,000 rpm for 15 min. Increased oil content decreased the droplet size such that value d_[90]_ subsequently reduced from 27.55, 25.90 to 20.31 µm in 15%, 30% and 45% rich emulsion, respectively. Within the emulsions, potentially, oil content might consequently increase with viscosity, thus preventing oil droplet collisions and thereby reducing flocculation at HIPE. The functional performance in many commercial food applications is often determined by rheological properties of emulsions such as viscosity, viscoelasticity, yield stress or elastic modulus (McClements, 2015). In this current work, Fig. 4 shows the storage and loss moduli of HIPE measured in stress sweep test. The increase of oil concentration led to a greater apparent viscosity up to 1.74 (±0.08) Pas in HIPE, which showed a shear thinning behavior with apparent viscosity reducing with increasing shear rate. The shear thinning property of HIPE was due to the molecules untangle as a consequence of orientation in flow direction accelerating a change in the effective volume (McClements, 2000). Considering that as rapeseed oil content increased with viscosity, it decreased mean droplet size. The apparent viscosities of LIPE and MIPE would depict a low value and remained a constant with increasing shear rate, indicating their Newtonian property. LIPE and MIPE had an apparent viscosity of 0.004 (±0.002) and 0.019 (±0.000) Pas, respectively at shear rate of 300 s^-1^. Elsewhere, Hebishy et al. (2017) manufactured 10%, 30% and 50% oil rich emulsions stabilized by whey protein isolate using conventional homogenization or ultra-high pressure homogenization (UHPH). Somewhat resembling the current findings, these 10, 30% and 50% lipids rich emulsions from Hebishy et al. (2017) obtained low viscosity and Newtonian behavior when emulsions were fabricated by conventional homogenization. However, the application of UHPH led to a formulation of resembling emulsion 50% oil with either shear thinning behavior or pseudo-plasticity, which simultaneously reduced the oil droplet size compared to those of conventional produced emulsion. Indeed, the homogenization conditions might have contributed to both droplet size and degree of interaction between oil (droplets) within the produced emulsions. Bellalta et al. (2012) reported stability of oil-in-water emulsions by whey protein wherein 50–55% oil rich emulsion obtained Newtonian behavior, but a further increase to 60% switched the rheological behavior to shear thinning. Thus, low oil emulsions should possess far apart droplets with relatively weaker interaction. As the concentration of oil increases, the droplets density increases with shortened mean distance between droplets, making London-van der Waals forces of attraction to become dominant. Oil droplets within the volume fraction would bring about inter-droplet interaction and collisions (Hebishy et al., 2017). In this work, the flow curves were also examined in all emulsions using three gaps at 0.30, 0.50 and 1.00 mm between cone-plate geometry. No thixotropic behavior occurred at LIPE and MIPE, but only HIPE with hysteresis of 1.60 (±0.10) J. Emulsion structure broke down while shearing, which seemed followed by a gradual recovery whilst reducing shear rate. The oscillation shear rheology of HIPE confirmed its viscoelastic behavior (Fig. 4). Data was fitted to Ostwald de Waele model at r^2^ 98%, the consistency index K resulted in 135.0 (±21.68) and a flow index n of 0.43 (±0.04). This result agrees with Wijaya et al. (2017) who evaluated the whey protein stabilized HIPE and characterized as viscoelastic gel-like systems.

**Table 1:** Characteristics of droplet size of LIPE, MIPE and HIPE.

Sample ID	d_[10]_(µm)	d_[50]_(µm)	d_[90]_(µm)	d_[3,2]_(µm)	Span
LIPE	1.4 (±0.30)	14.7 (±5.34)	36.0 (±9.73)	17.0 (±13.39)	2.35
MIPE	4.6 (±0.72)	8.8 (±1.63)	16.1 (±3.30)	9.3 (±4.54)	1.31
HIPE	4.3 (±0.71)	7.6 (±1.41)	12.3 (±2.33)	5.5 (±2.53)	1.04

**Note:**

Results are expressed as mean ± standard deviation. LIPE, low internal phase emulsion; MIPE, medium internal phase emulsion; HIPE, high internal phase emulsion.

**Table 2:** Characteristics of droplet size in yogurts.

Sample ID	Content of lipids (%)	D_[10]_(µm)	D_[50]_(µm)	D_[90]_(µm)	D_[3,2]_(µm)	Span
Emulsion yogurt	2.5%	2.6 (±0.30)	5.3 (±1.06)	8.7 (±1.82)	5.5 (±2.62)	1.14
Regular yogurt	2.5%	1.8 (±0.18)	3.3 (±0.43)	6.2 (±1.35)	3.4 (±1.61)	1.36
Fat free yogurt	0.0%	2.3 (±0.39)	2.8 (±0.42)	4.1 (±0.59)	2.8 (±0.80)	0.64

**Note:**

Results are expressed as mean ± standard deviation.

### Emulsions stability

**Figure 5:**
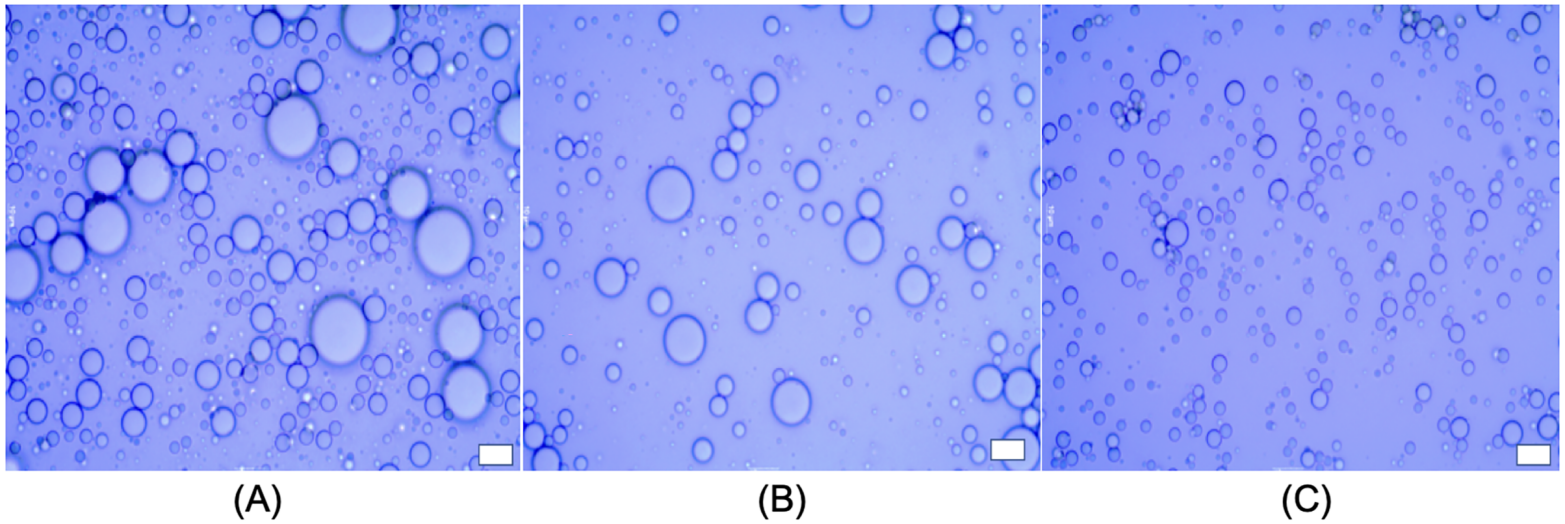
Microstructure of emulsions; LIPE (A), MIPE (B) and HIPE (C). Scale bar: 10 µm. LIPE, low internal phase emulsion; MIPE, medium internal phase emulsion; HIPE, high internal phase emulsion.

As whey proteins are commonly used ingredients in food colloids for thickening, gelation foaming and emulsification, the innovative application of whey proteins as applications in food industry has been continuously developing. As a number of studies confirmed the whey protein is an excellent emulsifier (Hebishy et al., 2017; Macierzanka et al., 2009; Zhao et al., 2021). Thus, the protein should help in designing new properties using chemical, enzymatic or physical modification, either alone or with other components/ingredients. In this current study, all physically structured o/w emulsions showed 100% encapsulation rate of rapeseed oil at day 30 of storage, indicating no loss of rapeseed oil by the centrifugation test (data not shown). During emulsification, the protein forms a protective membrane around the oil droplets, and simultaneously can reduce the interfacial tension (as shown in Fig. 5). The ζ-potential measured −28.60 ± 0.78, −30.40 ± 1.40 and 27.30 ± 0.83 mV for LIPE, MIPE and HIPE, respectively, which suggested whey protein above its isoelectric point, hence, appear strongly negatively charged, consistent with the opinion of Sarkar et al. (2009). Considering the microstructure of emulsions (as shown in Fig. 5), the formulated oil droplets showed some flocculation, irrespective of the incorporated content of oil. Probably, whey protein (such as beta-lactoglobulin) would adsorb at a neutral pH to o/w interface and stabilize the emulsion by strong electrostatic repulsion between the droplets (Dickinson, 2010). Post-absorption, however, the protein unfolds and oil droplets might approach each other towards flocculation, which depicts the formation of disulphide bonds between adsorbed molecules on different droplets and/or their hydrophobic association (McClements, 2004a). The phase separation calculated as creaming index indicated 41.2 (±1.79)%, 24.7 (±1.35)% and 0.0 (±0.00)% for 25%, 50% and 75% oil rich emulsions (data not shown). Although the creaming might not be necessarily considered as a criteria of emulsions instability (as this can be prevented by inclusion of viscosifiers), the droplet size might often remain at the same range during the emulsion storage with phase separation (Kasprzak et al., 2018). However, in this study we used the creaming as an instability factor, and thus as HIPE was resistant to phase separation, was further used as a carrier of encapsulated rapeseed oil to a stirred yogurt.

### Mean diameter and size distribution of oil droplets

**Figure 6:**
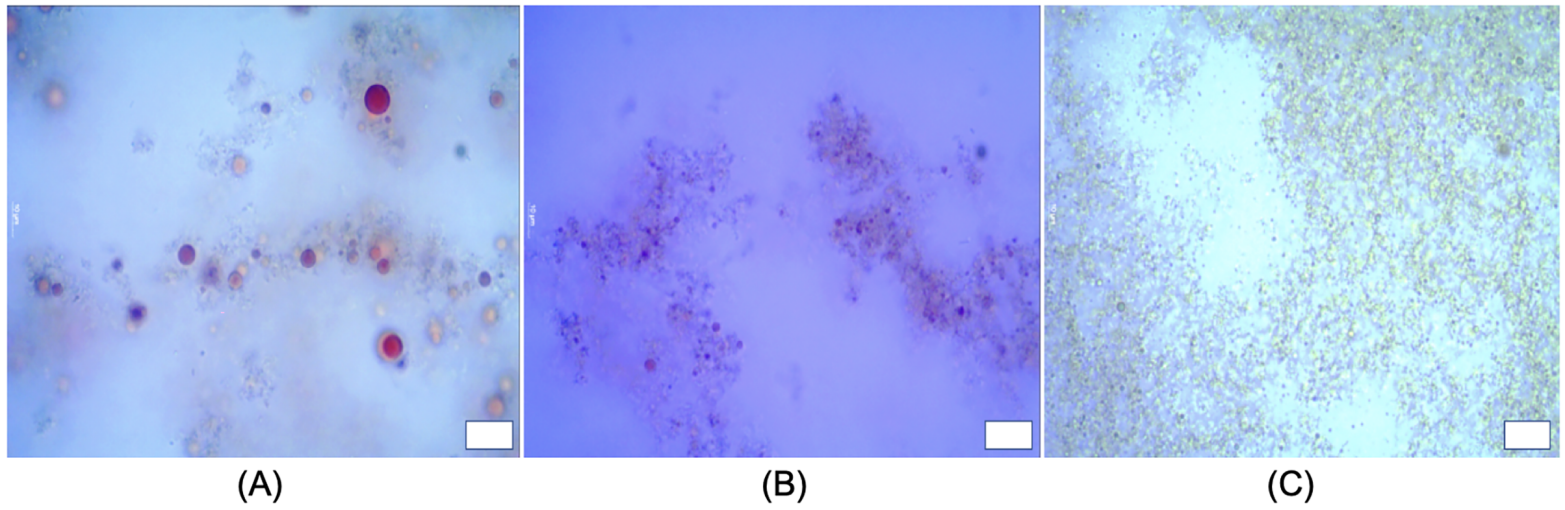
Microstructure of emulsion yogurt (A), regular (B) and fat free yogurt (C). Scale bar: 10 µm. HIPE, high internal phase emulsion.

**Figure 7:**
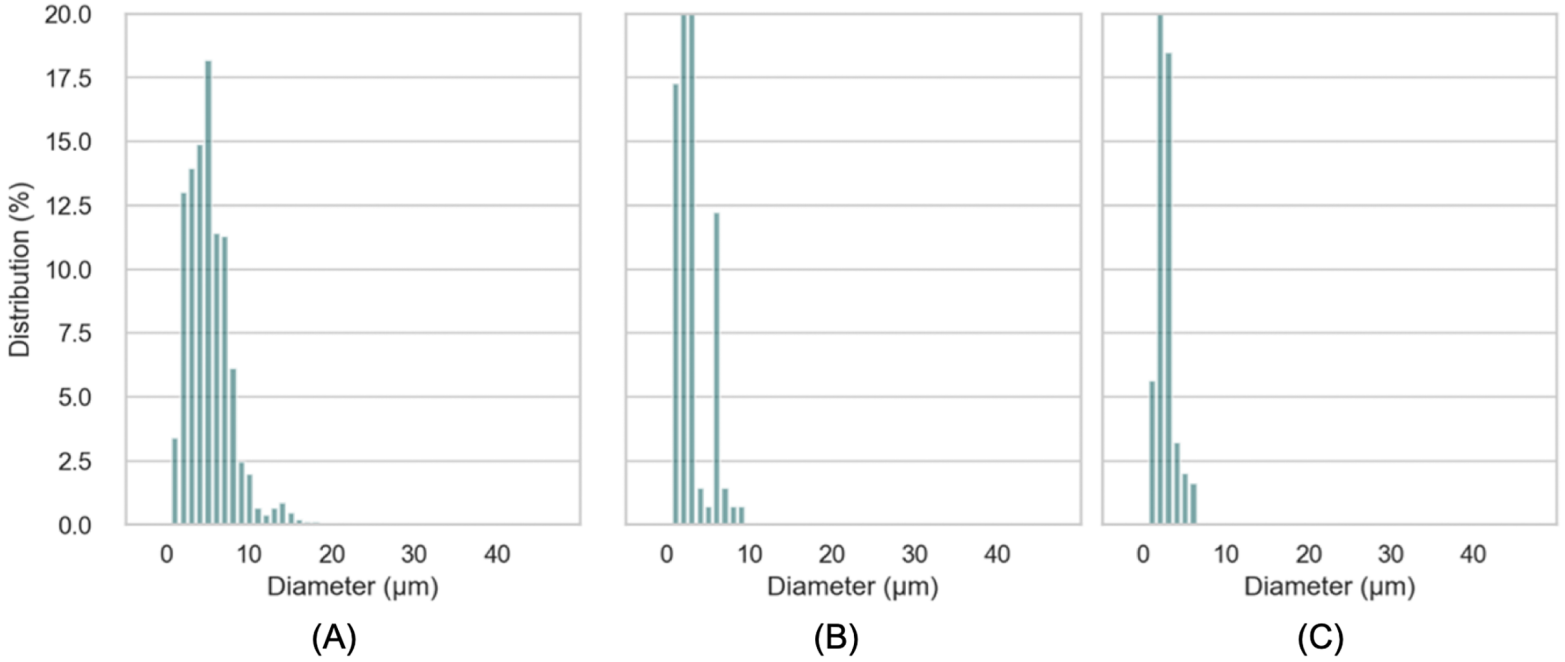
Distribution of droplet size in emulsion yogurt (A), regular yogurt (B) and fat free yogurt (C).

Substituting different ingredients in yogurt manufacture would influence protein composition/matrix structure (Karam et al., 2013). Typical yogurt manufacture modifies the milk properties irreversibly. The formulation of cream and milk powder leads to a standardization of the milk fat content in the final product (Damin et al., 2009; Peng et al., 2009; Tamime & Robinson, 2007). In this work, the oil droplets encapsulated with whey protein as a carrier, were incorporated into yogurt matrix. The presence of both oil droplets with protein particles (casein complexes) raise the issue for the determination of oil droplet size and oil droplets distribution in yogurts using the particle light scattering techniques. Figure 6 showed a presence of oil droplets marked on purple and casein complexes occurred as aggregates in yogurts. In order to assess the oil droplets morphology without interfering protein particles, we detected the droplets *via* ilastil and CellProfiler^TM^ software, followed by data analysis. The droplet size distribution in emulsion yogurt, regular yogurt and fat free yogurt, were presented in Fig. 7. Mean size of oil droplets present in emulsion yogurt seemed slightly decreased compared with 75% rapeseed oil emulsion alone. Similarly, the D_[10]_, D_[50]_ and D_[90]_ values decreased, whereas the Span value increased. Thickness or charge are the interfacial properties can reflect the nature of interaction between the droplets and protein particles (McClements & Jafari, 2018). Partially droplet shrinkage of HIPE in emulsion yogurt might be due to electrostatic interaction/charge in a new food matrix. In this current work, the values of pH recorded 4.1, 4.0 and 4.1 for emulsion yogurt, regular and fat free yogurt, respectively. Also, the ζ-potential recorded −11.5 ± 1.26, −7.0 ± 0.54 and −2.9 ± 0.93 mV for emulsion yogurt, regular and fat free yogurt, respectively. The highest value of ζ-potential in HIPE rich yogurt might suggest that the whey protein stabilized emulsion provide a slightly better stability of matrix system than milk protein stabilized milk fat itself. Whey protein is a mixture of globular milk proteins such as beta-lactoglobulin, alpha-lactalbumin, bovine serum albumin and immunoglobulins (Ravindran et al., 2018). The bovine casein is composed of individual casein fraction namely αS1-, αS2 -, β-, and κ-CN (Walstra, 1990). Depending on the pH and protein concentration, the isoelectric point of whey protein ranged between 4.8 and 5.4 (Deeth & Bansal, 2018), whereas the casein is about 4.6 (Carr & Golding, 2016). As pH yogurt approach the isoelectric point of whey protein, the process of changing the charge of emulsion stabilized whey protein/casein might be responsible for the gradual deprotonation of carboxyl and amino groups, which might reduce the overall impact of oil droplet interphase/ emulsion size. In testing the whey stabilized HIPE, Zhou, Drusch & Hogan (2022) found a greater stability of emulsions occurred with increased inter-droplet interactions at pH 3 compared to the neutral pH. The pH value, moreover, influenced the reduced or increased droplet size of emulsions, depending on the processing methods. The pH values of all tested yogurts were slightly below the isoelectric point of casein or whey protein. More so, increase in the calcium content in the mixture can further reduce the zeta potential of casein micelles (Post et al., 2012). The incorporation of HIPE might be an additional consequence of suppression of electrochemical charge in the yogurt product.

### Color and texture aspects

The impact of rapeseed oil content and yogurt type on the overall color of samples was characterized by reading their L*, a* and b* values using a colorimeter. Color of emulsions and yogurts can be seen in Table 3. Besides significant differences between the samples (*p* < 0.05), the lightness between the fabricated emulsions was similar. However, a* and b* values were subsequently reduced (*p* < 0.05) towards greener and less yellow, respectively with increasing content of rapeseed oil and reduce size of oil droplets. Notably, the emulsions being lighter and greener, and even more blue as droplet concentration increased (Chantrapornchai, Clydesdale & McClements, 1998) explains the behavior of the scattering and absorption of light by emulsions. The scattering efficiency of droplets would decrease with larger droplets, which suggests the light beam to penetrate further into the emulsions that result to increased absorption. Each emulsion was furthermore compared against one another, which accounted for a ΔE result of ≤3.0. The white color of yogurt or milk is a result of the light dispersion of milk fat globules and casein micelles (Kiełczewska et al., 2020). In the yogurt samples, fat free yogurt had slightly lower lightness compared to regular yogurt. However, the inclusion of HIPE into the fat free yogurt (formulating emulsion yogurt) raised the L value and reduced both a* and b* value, compared to fat free yogurt alone. The ΔE between emulsion yogurt, fat free and regular yogurt resulted in ≤1.9 (±0.08). A value of ΔE below 2 is often considered to be an unrecognizable for color difference by consumers (Maung et al., 2020). Minor changes in color proved that the inclusion of capsulated rapeseed oil able to enrich the liquid food with no compromise to the visual appearance. Yogurt texture is one of the most important attributes associated with overall quality. Any modification of the traditional fabrication method may lead to quality deterioration consequently changing consumers acceptance (Lesme et al., 2020). Incorporating emulsion after the fermentation process is a simple approach of delivery of component of interest, which should fully maintain within the product matrix. The textural attributes of emulsion yogurt, fat free and regular natural yogurt were characterized at temperature of 4 (±1) °C by compression test using a backwards extrusion. Table 4 shows the texture parameters of yogurts of this current work. The firmness of emulsions resulted in 0.2N, irrespective of yogurt type. The addition of emulsion into yogurt allowed for the whey protein (as an emulsifier of o/w system) to be incorporated, which appeared unable to change the texture (of emulsion rich yogurt). This situation appears to contrast other workers (Lesme et al., 2019, 2020) that demonstrated inclusion of protein resulted in differences in physicochemical properties. For emphasis, firmness is defined as the necessary force to attain a given deformation (Mousavi et al., 2019). Moreover, cohesiveness/consistency depicts the strength of inner bonds within the yogurt, which keeps the product’s structure (Mudgil, Barak & Khatkar, 2017). Resemblances in cohesiveness/consistency between the emulsion yogurt, regular or fat free yogurt, herein, suggests the whey protein (carrier) could deliver the encapsulated rapeseed oil into the natural yogurt (structure) without any influence on texture.

**Table 3:** Color of emulsions and yogurts.

Color	a*	b*	L
LIPE	−0.4 (±0.01)^a^	6.1 (±0.02)^b^	80.7 (±0.04)^e^
MIPE	−0.6 (±0.02) ^b^	5.1 (±0.01)^c^	83.0 (±0.03)^a^
HIPE	−0.9 (±0.07) ^c^	3.5 (±0.37)^d^	82.0 (±0.24)^c^
Emulsion yogurt	−2.1 (±0.02) ^e^	6.8 (±0.01)^a^	81.4 (±0.08)^d^
Regular yogurt	−1.9 (±0.01)^d^	7.0 (±0.00)^a^	82.4 (±0.02)^b^
Fat free yogurt	−2.4 (±0.01)^f^	6.9 (±0.02)^a^	80.6 (±0.09)^e^

**Note:**

Values in the same column with different letters indicate significant differences in emulsions or yogurts group, according to ANOVA (*p* < 0.05). Results are expressed as mean ± standard deviation.

**Table 4:** Texture parameters of yogurts.

	Firmness	Cohesiveness	Consistency
Emulsion yogurt	0.2 (±0.01)	−0.2 (±0.01)	4.9 (±0.01)
Regular yogurt	0.2 (±0.02)	−0.2 (±0.01)	5.2 (±0.64)
Fat free yogurt	0.2 (±0.01)	−0.1 (±0.01)	4.8 (±0.10)

**Note:**

Values are expressed as mean ± standard deviation. There were no significant differences in measured parameters (*p* value > 0.05).

### Microbial aspects

Many factors could affect the viability of starter bacteria in yogurts, for instance, the composition of yogurt mix, especially in the context of non-dairy components. Further, the processes of either filling and/or mixing remain of great importance, which would result in the presence of oxygen in the emergent product that negatively affect the viability of bacteria in the yogurt (Talwalkar & Kailasapathy, 2004). In order to verify whether the inclusion of emulsion might negatively impact the microbiological status of yogurts, the enumeration of *Streptococcus thermophilus* and *Lactobacillus delbrueckii* subsp. *bulgaricus* was determined before and after inclusion of emulsion in fat free yogurt. The microbiological parameters of emulsion, regular and free-fat yogurt are shown in Table 5. Interestingly, the number of *Streptococcus thermophilus* resulted in 9.28 ± 0.07, 9.28 ± 0.06 and 8.82 ± 0.08 Log cfu/g in fat free yogurt, regular and emulsion yogurt, respectively. Similarly, the number of *Lactobacillus delbrueckii subsp. bulgaricus* showed a value of 7.59 (±0.06), 8.00 (±0.08) and 7.57 (±0.03) Log cfu/g in fat free yogurt, regular and emulsion yogurt, respectively. This suggests that the inclusion of oil rich emulsion into the natural drinking yogurt would likely not reduce the bacterial viability. According to Standard for Fermented Milks (CXS 243-2003) the sum of *S. thermophilus* and *L. delbrueckii* ssp. *bulgaricus* in yogurt samples should be of at least 10^7^ Log cfu/g. Based on the European Food Safety Authority (EFSA), the yogurt should contain at least 10^8^ CFU live starter bacteria per gram, in order to claim that the yogurt improves digestion of lactose. In addition, the amount of filamentous fungi and yeast and found values were verified as below 10 Log cfu/g across all yogurts. Moreover, microorganisms that often cause yogurt spoilage (Tamime & Robinson, 2007) when at such low level would indicate storage stability.

**Table 5:** Microbiological parameters of emulsion, regular and fat free yogurt.

	*S. thermophilus*	*L. delbrueckii* ssp. bulgaricus
	Log cfu/g	Log cfu/g
Emulsion yogurt	8.82 (±0.08)	7.57 (±0.03)
Regular yogurt	9.28 (±0.07)	7.59 (±0.06)
Fat free yogurt	9.28 (±0.06)	8.00 (±0.08)

**Note:**

Results are expressed as mean ± standard deviation.

### Sensory aspects

**Figure 8:**
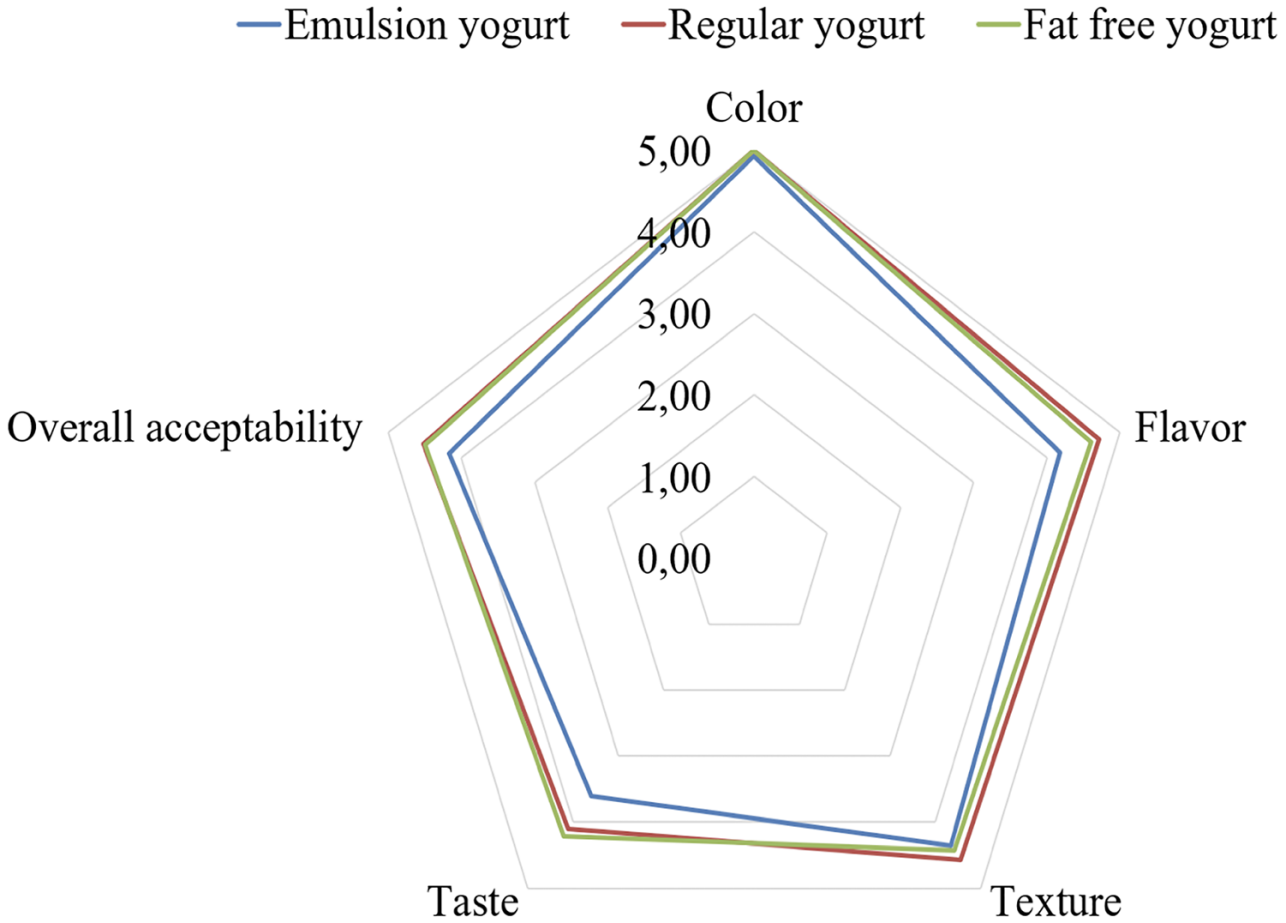
Overall acceptability of fat free, regular and emulsion yogurts.

Sensory evaluation has for long helped to complement product physicochemical properties, especially in the context of customer acceptability. In the current work, the three yogurt samples, namely fat free, regular and emulsion yogurt, were subjected to five-point sensory scale with respect to appearance, whey exudate and smell, texture, taste and overall acceptability. The appearance, whey exudate and smell evaluation showed resembling values (*p* > 0.05) of 5.0 (±0.00), 4.9 (±0.27) and 4.6 (±0.63) for fat free yogurt, 5.0 (±0.00), 4.9 (±0.27), 4.7 (±0.61) for regular yogurt and 4.93 (±0.27), 4.9 (±0.36) and 4.2 (±0.97) for emulsion rich yogurt, respectively. Similarly, values of texture, taste and overall acceptability were 4.4 (±0.76), 4.2 (±0.83) and 4.5 (±0.42) for fat free yogurt; 4.6 (±0.65), 4.1 (±0.63) and 4.5 (±0.33) for regular yogurt and 4.4 (±0.72), 3.6 (±0.79) and 4.2 (±0.4) for emulsion yogurt, respectively. Thus, although some numerical differences occurred, the inclusion of HIPE did not significantly affect the sensory property. Matrix of natural stirred yogurt could be an excellent base for small detection of foreign ingredients. In the current work, the appearance, smell and consistency showed resembling values (*p* > 0.05) of 8.00 ±0.95), 7.50 (±1.90) and 6.43 ±1.69) for fat free yogurt; 8.07 (±0.83), 7.29 (±1.20) and 6.43 (±1.83) for regular yogurt; 7.86 (±0.95), 6.07 (±1.90) and 6.07 (±1.69) for emulsion rich yogurt, respectively. Further, the taste and overall acceptability showed resembling values (*p* > 0.05) of 6.00 (±2.11) and 6.36 (±1.74) for fat free yogurt; 6.00 (±1.96) and 6.21 (±1.58) for regular yogurt; and 5.43 (±2.34) and 5.64 (±1.60) for emulsion rich yogurt, respectively. To provide a pictorial context, the overall acceptability of fat free, emulsion and regular yogurt samples, is shown in Fig. 8. Some study showed partial supplementation of milk fat with five vegetable oils (flaxseed, camelina, raspberry, blackcurrant and purple viper’s-bugloss) demonstrated yogurts as acceptable with some exceptions where off flavors emerged of raspberry and purple viper’s-bugloss (Dal Bello et al., 2015). Elsewhere, replacing milk fat with rapeseed or sesame oil in yogurts would show overall sensory scores of rapeseed or sesame oil rich yogurts to resemble control milk fat yogurt (Farmani et al., 2016). By characterizing the yogurts enriched walnut and flaxseed oil, the milk fat control resulted with highest overall acceptability (Baba et al., 2018).

## Conclusions

To our best knowledge, this is the first study that demonstrated the feasibility of simple manufacture of HIPE with the incorporation into the natural stirred yogurt as a milk fat replacement approach. The main conclusions could be stated as follows:

1. Successfully produced emulsions were stable without oil loss for 30 days at 4 °C, irrespectively of added content of rapeseed oil.
2. Size of oil droplets was influenced by the content of rapeseed oil.
3. The size of oil droplet led to a reduction when HIPE was incorporated into the yogurt matrix due to a change of pH and/or additional homogenisation.
4. The textural and organoleptic properties and microbiological status of the emulsion yogurt were similar with fat free or regular yogurt.

In the course of food product development, selected plant-based lipids might be encapsulated by whey protein and added into liquid or semi-liquid foods in order to induce a potential health benefit.

## Supplemental Information

10.7717/peerj.16441/supp-1 Supplemental Information 1 Raw data.

## Additional Information and Declarations

### Data Availability

The following information was supplied regarding data availability: The raw data are available in a Supplemental File.
