# Supplementary figures and images for: Replacement of milk fat by rapeseed oil stabilised emulsion in commercial yogurt
Source: PeerJ. 2023 Dec 11;11:e16441. doi: 10.7717/peerj.16441 (PMC10720406; doi:10.7717/peerj.16441)

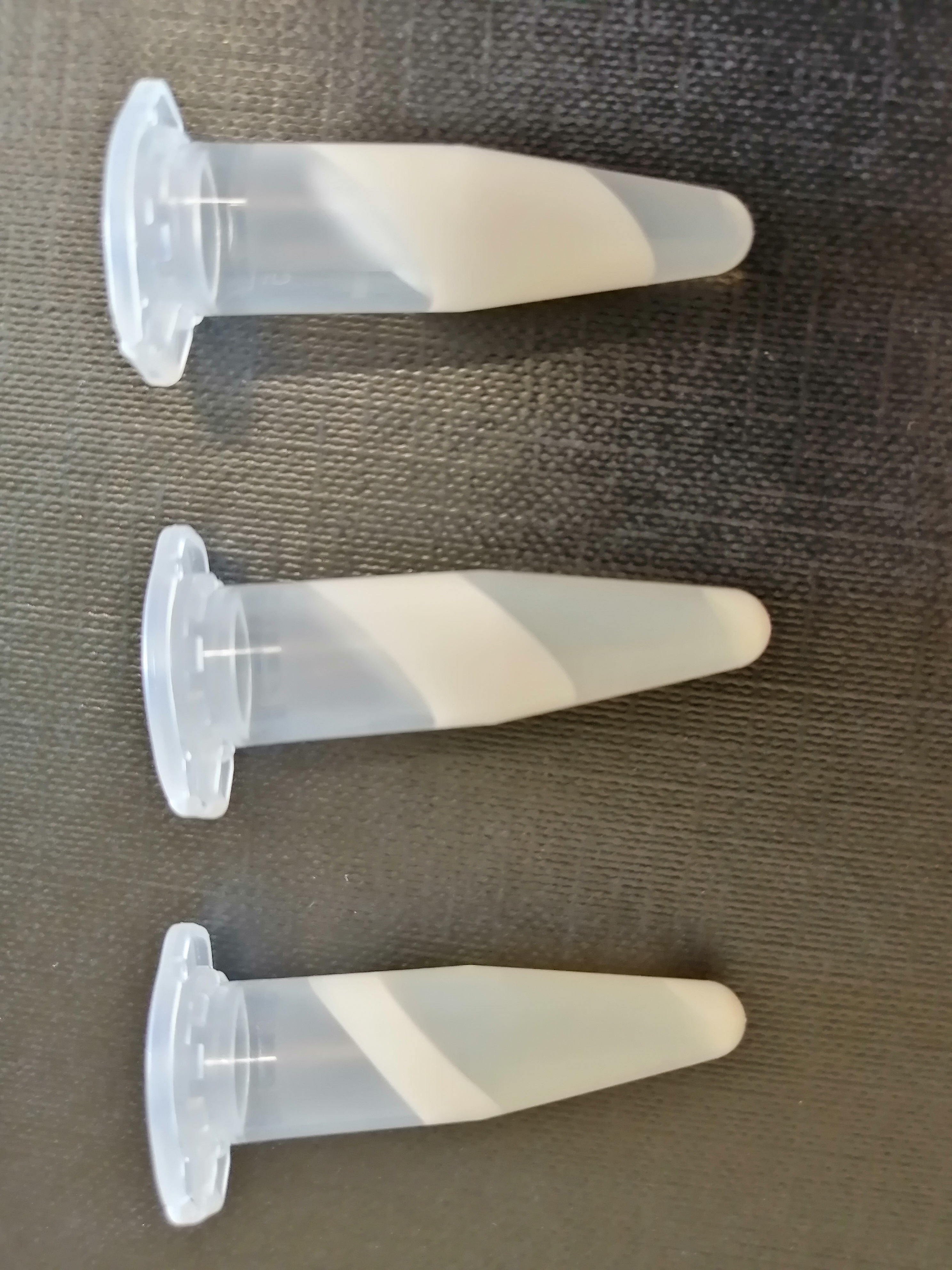

Supplement: Supplemental Information 1 [file peerj-11-16441-s001.zip › encapsulation of all emulsions_100%.jpg]
